# Microangiopathic Anemia of Acute Brucellosis – is it a True TTP?

**DOI:** 10.4084/MJHID.2010.031

**Published:** 2010-10-02

**Authors:** Amir A Kuperman, Amjad Baidousi, Maher Nasser, Andre Braester, Faris Nassar

**Affiliations:** 1 Hematology Institute, Western Galilee Hospital, Naharia; 2 Department of Medicine “E”, Western Galilee Hospital, Naharia, Israel Affiliated with the Bruce Rappaport Faculty of Medicine, Israel Institute of Technology, Haifa, Israel

## Abstract

Thrombotic thrombocytopenic purpura (TTP) is a severe disease, potentially fatal, if not diagnosed and treated promptly. TTP is clinically characterized by the pentad of thrombocytopenia, Coombs-negative hemolytic anemia, fever, renal abnormalities and neurological disturbances. Advances in recent years have delineated the molecular mechanisms of acquired and hereditary TTP.

Many infectious organisms have been reported to be associated with TTP, especially *mycoplasma*, but few cases of *Brucella* infection associated with thrombotic microangiopathy have been reported.

We describe a young woman who presented with TTP after acute infection with both *Brucella melitensis* and *Brucella abortus*. The patient completely recovered following aggressive therapy with plasmapharesis, high-dose corticosteroids and appropriate antimicrobial therapy.

Since measurement of ADAMTS13 activity and neutralizing antibodies is now available, and none of the reported cases of brucellosis with thrombotic microangiopathy (including the present report) were tested, for better understanding of this rare association, we recommend this work-up in future cases.

## Introduction:

Thrombotic thrombocytopenic purpura (TTP) was first described by Moschowitz in 1925.^1^ Since then, various infections have been reported to be associated with TTP. Acute Brucellosis is rarely associated with TTP, and has been reported only in a few cases.^2^–^7^

## Case report:

A 27 year-old previously healthy female, was admitted after three days abdominal pain, vomiting, headache, general malaise, and fever since the night prior to admission. Two weeks earlier, the patient noticed red urine and excessive gum bleeding during teeth brushing. No medications were used. Physical examination revealed low grade fever of 37.8°C, blood pressure 121/54 mmHg, and a regular heart rate of 54/min. There were some purpura on her legs, back and breast, a moderate splenomegaly and mild jaundice. Fundus examination was normal.

Laboratory tests at admission showed hemoglobin: 9.9 g/dl, white blood cells: 5.4 x10^9^/L, platelets: 7 x10^9^/L, and reticulocyte counts: 2.7%. Peripheral blood smear presenteded red blood cell fragmentation (schistocytes).

Lactate dehydrogenase was 3,046 IU/L, aspartate aminotransferase 153 IU/L, gamma-glutamyl transferase 39 IU/L, alkaline phosphatase 138 IU/L, total bilirubin 6.6 mg/dl, direct bilirubin 2.5mg/dl. Blood urea nitrogen and creatinine were within normal limits.

Urinalysis showed hematuria and hemoglobinuria. Coagulation screening tests were normal, fibrinogen degradation products (FDG) were increased (over 492mg/dl). Bone-marrow aspirate showed active hematopoiesis without blasts.

Viral serology tests, including *hepatitis B virus*, *hepatitis C virus*, *Epstein-Bar virus*, *cyto-megalo virus* and *parvo virus* were all negative, as well as anti-nuclear antibodies, anti double-stranded DNA, anti Sm and anti RNP. C3 and C4 levels were normal. Chest x-ray was normal, while abdominal ultra-sound showed moderate splenomegaly.

A diagnosis of TTP was made, and treatment with intravenous methylprednisolone was started, but the hemoglobin continued to drop to levels of 5.9 mg/dl without significant improvement in the platelet count, which entitled red blood cell and platelet transfusions. Plasma-exchange was performed for 9 days with significant improvement in platelets and hemoglobin levels.

On the 8^th^ day after admission, blood culture yielded *Brucella. Brucella* agglutination titer was positive for two strains: 1/320 for both *Brucella melitensis* and *Brucella abortus*. Blood culture was repeatedly positive for *Brucella*. A second *Brucella* agglutination titer was 1/1,280 for Brucella melitensis and 1/640 for *Brucella abortus*. Antibiotic treatment with doxycycline and gentamicin was commenced.

**Figure 1.**
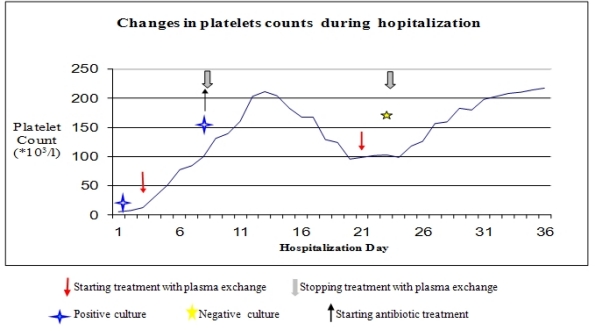
Platelet counts during treatment

After platelet counts recovered, plasma-exchange was stopped, but platelets gradually dropped to 96 x10^9^/L in the following week, without significant changes in the hemoglobin level. Plasmapheresis was resumed on alternate days, with recovery of platelet counts to normal levels. Follow-up blood cultures and *Brucella* serology turned negative (figure 1).

## Discussion:

Brucellosis is a major health problem in many parts of the world, particularly in the Mediterranean and the Middle East countries. It is a multisystem disease with a broad spectrum of clinical manifestations. Hematological abnormalities ranging from a fulminant state of disseminated intravascular coagulopathy to subtle hemostatic alterations have been reported in *Brucella* infection. Mild anemia and leucopenia have been frequently associated with acute brucellosis, but pancytopenia and thrombocytopenia are less frequent. Thrombocytopenia during the clinical course of brucellosis presents an incidence varying from 1% to 8% of cases in adults.^8^

The clinical picture of TTP consists of a prodromal phase, a virus-like disease occurring in up to 40% of patients. About 60% of patients have neurologic disturbances, 90% initially have purpura and fever is constantly observed. Anemia is often severe with hemoglobin levels of 7–9 g/dl, renal involvement is present in 90%, with renal failure in 40–80% of patients.

Advances in recent years have delineated the molecular mechanisms of acquired and hereditary TTP and many of the atypical cases of Hemolytic Uremic Syndrome (HUS). These studies have clearly demonstrated that the syndrome of thrombotic microangiopathy encompasses several distinct molecular defects. TTP has been recognized to result from a profound deficiency of the VWF (von Willebrand factor) cleaving metalloprotease ADAMTS13. Up to 50% of the cases of atypical HUS have been associated with complement dysregulation, due to mutations or autoantibodies of complement factor H, serine protease factor I or membrane cofactor protein CD46.^9^ Although each disorder may present with the characteristic constellation of features, there is substantial overlapping, making it difficult to distinguish these disorders solely based on clinical manifestations. Clinical diagnosis of TTP and HUS not based on pathophysiology or molecular defect is quite variable and has contributed to confusing literature.

Until the introduction of plasma exchange therapy in the 1970’s, TTP was almost universally fatal. Plasmapheresis is considered the mainstay of therapy, removing unusually large VWF (UL-VWF) and antibodies against the VWF cleaving metalloprotease on one hand, and replenishing the metalloprotease levels on the other.

Bell et al reported on the efficiency of this treatment that led to a notable decrease in mortality rates from near 100% to less than 10%.^1^

Among other causes of TTP, various infections have been implicated, although an infectious agent has not been always isolated. Other communications have emphasized the association between rising titers of *coxsackie B* and *CMV* infection with *Mycoplasma pneumoniae*, *Mycobacterium tuberculosis*, *Borrelia burgdorferi*, *Herpes zoster*, *Legionella pneumophilia*, *Bacteroides*, vaccination against *influenza* and *typhoid fever type A and B* and a concurrent presentation of TTP.

Review of the published literature revealed at least 6 cases with brucellosis-associated thrombotic microangiopathy (Table 1).

Five cases occurred in urkey and 1 in Italy suggesting that brucellosis is endemic in the Mediterranean region. Common clinical features in all reported cases were anemia, thrombocytopenia and schistocytes on blood smear, while neurologic symptoms and renal insufficiency were less frequent. In none of these published cases had ADAMTS-13 activity or neutralizing antibodies been performed. Four of the patients had been treated without plasmapheresis, and 3 of them survived. The lack of renal and neurologic symptoms, and the high survival rate for TTP without plasmapheresis, raises the question: “Is it a true TTP?”.

The patients described had the features of microangiopathic hemolytic anemia, and the differential diagnosis was TTP or disseminated intravascular coagulation (DIC). Normal coagulation tests (prothrombin time, activated partial thromboplastin time, fibrinogen and fibrinogen degradation products) excluded the possibility of DIC. Unfortunately, at that time the assay for ADAMTS13 activity and neutralizing antibodies was not available, though it is today.

In summary, we present a case of a young woman who presented with TTP after an acute illness with concomitant infection of two *Brucella* strains: *Brucella melitensis* and *Brucella abortus*. We hypothesize that cross-reactive antibodies between *Brucella* antigens and the VWF-cleaving metalloprotease or direct assault of the endothelium by the bacteria are the basis to this rare association. Prompt treatment with plasma exchange is the mainstay for therapy and is indeed life-saving. As in other cases of refractory or exacerbated TTP, treatment of the infection is mandatory in order to establish a rapid and solid remission.

## Figures and Tables

**Table 1:** Summary of reported cases.

Age (years)	Gender	Country	Evidence for *Brucella*	Clinical features	ADAMTS-13	Therapy	Outcome	Reference
19	Male	Italy	Blood culture	A, T, S	ND	Ab	AW	2
11	Female	Turkey	Serology, BM calture	A, T, S	ND	Ab	AW	3
71	Male	Turkey	Serology, blood calture	A, T, S, N	ND	Ab, plasmapheresis	AW	4
51	Male	Turkey	Serology	A, T, S, N, R	ND	Ab, MPD, FFP	AW	5
19	Female	Turkey	Serology, BM calture	A, T, S, N	ND	Ab, FFP, plasmapheresis	AW	6
7	Female	Turkey	Serology,	A, T, S	ND	Ab, MPD, FFP	death	7

Ab–Antibiotics, N–Neurological symptoms, MPL–Methylprednisolone, S–Schiztocytes, FFP–Fresh Frozen Plasma, ND–Not Done, A–Anemia, AW–Alive and Well, T–Thrombocytopenia, R–Renal Insufficiency
